# Clinicians’ Experiences of Implementing Clinical Frailty Scale Assessments in Lung Oncology Clinics: A Qualitative Interview Study

**DOI:** 10.3390/cancers18050884

**Published:** 2026-03-09

**Authors:** Jessica Pearce, Hayat Hamzeh, Mary Denholm, Alastair Greystoke, Fabio Gomes, Andrew Clegg, Galina Velikova, Suzanne H. Richards, Alexandra Gilbert

**Affiliations:** 1 Leeds Institute of Medical Research, University of Leeds, Leeds LS2 9JT, UK; 2 Leeds Institute of Oncology, Leeds Teaching Hospitals NHS Trust, Leeds LS9 7TF, UK; 3 Department of Oncology, Cambridge University Hospitals NHS Foundation Trust, Cambridge CB2 0QQ, UK; 4 Northern Centre for Cancer Care, Newcastle upon Tyne Hospitals NHS Foundation Trust, Newcastle upon Tyne NE7 7DN, UK; 5 Senior Adult Oncology Team, The Christie National Health Service Foundation Trust, Manchester M20 4BX, UK; 6 Academic Unit for Ageing and Stroke Research, Bradford Institute for Health Research, University of Leeds, Bradford BD9 6RJ, UK; 7 Leeds Institute of Health Sciences, University of Leeds, Leeds LS2 9NL, UK

**Keywords:** frailty, geriatric oncology, qualitative research, frailty assessment, frailty screening, clinical frailty scale (CFS), frailty-informed care

## Abstract

Background/Objectives: Simple frailty assessments, such as the clinical frailty scale (CFS), are prognostic for worse outcomes in older adults with cancer and could support treatment decision-making. This interview study aims to explore clinicians’ experiences of using simple frailty assessments in oncology, including the impacts on patient care and barriers and facilitators to successful implementation. Methods: Semi-structured individual interviews were conducted with clinicians at three UK sites that had implemented CFS screening in lung cancer clinics as part of a national pilot, to explore how frailty assessments are applied and are impacting care. Purposive sampling targeted a range of professionals involved in assessing frailty and making treatment decisions. Recordings were transcribed verbatim and analysed thematically. Results: Ten clinicians participated, and four main themes were identified. ‘Assessing fitness and frailty’ explores the central role of performance status (PS), as well as its limitations, and what frailty assessments add. ‘Scoring and interpreting CFS’ describes the ease and relative yield of CFS use, particularly for patients with ‘borderline’ PS scores (e.g., PS 1–2 or 2–3), and the importance of contextual interpretation. ‘Role of frailty and impacts of assessment’ highlights how frailty assessments can enhance patient-centered care and support, and clinical and shared decision-making, with potential for streamlined care and system-level benefits. ‘Barriers and facilitators to implementation’ are described, including time, culture, guidance, and training, with recommendations provided. Conclusions: Assessing frailty has wide-ranging potential benefits for patients, oncology teams, and the wider system, but barriers must be overcome. Specific recommendations are provided to support the routine implementation of frailty assessments, which is a key step towards the benefits of frailty-informed care being realised at scale.

## Simple Summary

Simple frailty assessments, such as the clinical frailty scale (CFS), could support treatment decision-making and care in cancer clinics, but they are not currently used routinely. This qualitative interview study explored clinicians’ experiences of using frailty assessments in lung cancer clinics to understand how they impact care, and the barriers and facilitators to their use. Four main themes were identified. ‘Assessing fitness and frailty’ explores the central role of performance status in assessing fitness and accessing cancer treatments, as well as its limitations and what frailty assessments add. ‘Scoring and interpreting CFS’ describes the ease and relative yield of CFS use, and its ability to differentiate between patients considered ‘borderline’ according to performance status, as well as the need to consider scoring in the wider clinical context. ‘Role of frailty and impacts of assessment’ highlights how frailty assessments can enhance patient-centred care and support, communication with patients, and clinical and shared decision-making, with the potential to streamline care and convey wider system-level benefits. ‘Barriers and facilitators to implementation’ describes factors that help or hinder the delivery of frailty assessments and frailty-informed care, with specific recommendations provided to support use in practice.

## 1. Introduction

It is increasingly recognised that performance status, which is widely used to assess fitness for systemic anti-cancer treatment (SACT), lacks granularity for evaluating complex and older patients [1]. Frailty is a common condition that is associated with ageing and is characterised by reduced physiological reserves and vulnerability to worse outcomes. Various measures of frailty consistently provide useful prognostic information across a range of clinical populations and outcomes, including in people with cancer [2,3].

International guidance recommends that older adults being considered for SACT undergo geriatric assessments to evaluate various domains contributing to frailty (e.g., function, nutrition, cognition) [4,5,6,7]. However, geriatric assessments can be time and resource-intensive, generally requiring trained staff. These barriers to implementation mean they are not available to most patients [8,9]. Brief frailty screening tools provide a potential pragmatic solution with a dual purpose: to improve on routine assessments to support decision-making about cancer treatment and to identify potentially vulnerable patients who may benefit from a more in-depth geriatric assessment and optimisation. The rationale for using frailty screening tools to support SACT decision-making is supported by a 2025 meta-analysis demonstrating that even brief frailty tools are prognostic for a range of important outcomes in this population, including mortality, severe toxicity, treatment intolerance, and hospitalisation [3]. It is increasingly recognised that healthcare decisions should be made together with patients. In the UK, guidance was published by the National Institute of Clinical Excellence (NICE) in 2021 promoting shared decision-making [10], a collaborative process in which patients and healthcare teams make decisions together, and various national bodies suggest frailty assessments should have a key role in this process [11,12].

The clinical frailty scale (CFS) is a 9-point clinician-assessed scale based on health-related functioning [13], which can be undertaken in minutes [14] and is prognostic in cancer populations [15,16]. Five sites in the United Kingdom (UK) began piloting routine use of CFS to screen for frailty in lung cancer clinics in 2018 as part of a national quality improvement initiative led by the Specialised Clinical Frailty Network (SCFN) and commissioned by NHS England [12]. Frailty assessments may be considered particularly relevant in the lung cancer context, where patients are commonly diagnosed at an advanced stage and often have co-morbidities contributing to frailty. Local evaluation at two SCFN pilot sites provided early evidence of the acceptability and feasibility of using CFS in oncology outpatient settings [12]. A 2024 qualitative study explored patient and staff perspectives on frailty assessment at a third SCFN site and provided valuable reflections on how frailty was described, assessed, and perceived. The analysis focused on key challenges of using frailty assessments in decision-making, including difficulties incorporating them in a way that is acceptable to patients and avoids potential unintended harms [17]. A more detailed exploration of the practicalities of implementing frailty assessments and their role in supporting treatment decision-making and wider care is warranted.

This qualitative interview study is being undertaken as part of the frailty-informed cancer management (FRAME) intervention development study [18] and aims to explore clinicians' experiences of using simple frailty assessments to inform cancer care, including the barriers and facilitators to successful implementation and potential impacts on patient care [18].

## 2. Materials and Methods

### 2.1. Ethics

Ethical approval was granted by the Leeds West Regional Ethics Committee (REC reference: 23/YH/0082), and institutional approvals were gained prior to recruitment commencing.

### 2.2. Design

A qualitative interview study was undertaken to allow in-depth exploration of clinicians’ experiences [19] of implementing frailty assessment at three NHS hospitals in the UK. The qualitative study was undertaken and reported in line with COnsolidated criteria for REporting Qualitative research (COREQ) guidance [20].

### 2.3. Underpinning Theory and Approach

A critical realist approach was adopted to understand individual experiences of frailty assessment within the broader social context of clinical practice. Two existing theoretical frameworks, Normalisation Process Theory (NPT) and the Making INformed Decisions—Individually and Together (MIND-IT) shared decision-making framework, informed the methods. NPT [21] describes four domains needed for successful implementation of interventions into routine work (coherence; cognitive participation; collective action; and reflexive monitoring) and was used to conceptualise the current and potential future implementation of frailty assessments in routine practice. MIND-IT [22,23] was used to explore experiences of shared decision-making within the consultation and how frailty assessments impact the process.

### 2.4. Setting/Site Sampling and Mapping Exercise

Three SCFN sites with varied local populations and service models were sampled (Cambridge University Hospitals NHS Foundation Trust (FT), The Christie NHS FT (Manchester), and Newcastle upon Tyne NHS FT). A mapping exercise was undertaken before the interview study commenced to gather preliminary contextual data about frailty assessment implementation across the three sites. This involved an interview-administered survey with frailty project leads (or a locally agreed representative) to assess service delivery [24]. Descriptions of service models from the mapping exercise informed participant sampling and topic guides for the interview study.

### 2.5. Qualitative Study Participant Sampling

Eligible clinicians included those involved in undertaking assessments and/or subsequent SACT decision-making; there were no exclusion criteria [Appendix A.1]. A purposive sample of 10–12 was sought, and recruitment continued until the sample was considered holding adequate information power to address the study aims [25].

Two purposive sampling techniques were combined [26]. Stakeholder sampling by professional role ensured different types of clinicians involved in assessing patients and utilising the information from assessments within cancer treatment decision-making were approached (e.g., physicians, clinical nurse specialists (CNS), and allied health professionals (AHPs)). Maximum variation sampling captured a diverse range of perspectives, with variation sought in terms of clinicians’ hospital site, grade, and self-reported adoption of frailty assessments.

### 2.6. Participant Approach, Recruitment, and Consent

The initial sampling and email approach was undertaken by site-specific principal investigators. Participation was voluntary, and no incentives were offered. Potential participants were instructed to respond directly to the research team to ensure participation and help maintain anonymity. Up to two reminder emails were sent.

Proportionate consent [27] was obtained via email by the lead researcher (JP), and demographic information (profession, grade, tumour site, and self-reported adoption of frailty assessments) was collected.

### 2.7. Data Generation

Semi-structured individual (one-to-one) interviews were conducted by the lead researcher (JP), a medical oncology registrar and PhD candidate with prior experience in qualitative methods. Interviews were conducted remotely via videocall (Microsoft Teams) in a private room, with no observers, within the participants’ working hours. No relationship was established with participants prior to study commencement. No participants withdrew.

Interviews followed a topic guide developed with input from the expert supervisory team (including two consultant oncologists [GV/AGi], a geriatrician [AC], and a qualitative methodologist [SR]) and informed by the mapping exercise. They were written using the MIND-IT framework [22,23] to explore experiences of shared decision-making within the consultation and NPT [21] to explore the implementation/integration of frailty assessments into routine work. The topic guide included an introductory paragraph outlining the interview focus and contextualising it within the wider intervention development study [18] [Appendix B.1]. Interviews explored how frailty assessment is implemented, whether and how it informs decision-making and care, and the barriers and enablers to implementing frailty assessments and shared decision-making within care planning. The primary focus was on the use and impact of frailty screening tools, particularly CFS, as opposed to more comprehensive/in-depth geriatric assessments. Demographic data were collected from participants to provide valuable contextual information and support purposive sampling. Field notes and a reflexive journal were completed by the lead researcher after each interview to supplement the analysis, and the guide was iteratively modified as the study progressed.

### 2.8. Data Analysis

Interviews were audio-recorded, transcribed verbatim, anonymised, and thematically analysed [28], aided by NVivo software (version 14) [29]. Transcripts were coded iteratively [30], with preliminary codes revised in light of the coding of subsequent transcripts and applied to all interviews. A framework approach was taken; early coding was deductive and aligned to MIND-IT [22,23] and NPT [21]. Thematic codes that did not fit the existing framework were coded inductively [31], allowing the identification of new concepts from the data. All transcripts were coded by the lead researcher [JP], and a third was coded independently [HH, a physiotherapist with prior qualitative experience in oncology] for analytical rigour. Using a series of meeting discussions and exchanging written reflections, the two reviewers followed a rigorous iterative process to revise the emerging themes and agree on subsequent coding [32]. At one single timepoint towards the end of the analysis, during the process of combing and condensing the codes, the generative Artificial Intelligence platform Microsoft co-pilot was used to provide inspiration for naming the groupings of key themes and sub-themes, which were reviewed and adapted by the reviewers [33]. The final structure and titles of the groupings and key themes presented were decided and agreed upon by the two reviewers. The last stage of the analysis involved the development of practical recommendations for implementation, which are presented within the four domains of implementation activity described (and mapped to NPT) by May et al. (information, empowerment, service user, and leadership strategies) [34]. Individual participant checking was not used, but feedback was sought on the final report from clinicians at each site (including MD/AGr/FG). Positional reflexivity, and the impact of the lead researcher's professional role and pre-existing assumptions as an oncologist with an interest in frailty, were considered during analysis [35] and balanced by the involvement of the wider research team.

## 3. Results

### 3.1. Key Contextual Information

Mapping undertaken with leads from each site highlighted similarities and variations in how CFS screening had been implemented. All sites initially promoted CFS screening in the lung cancer setting for patients of any age and eventually integrated it into their electronic health records (EHRs). Initial goals of CFS implementation included documenting rates of frailty and local need, allowing identification and optimisation of frail patients, and supporting decision-making. Two sites (The Christie and Newcastle) used data from the initial pilot to inform the development of, and obtain funding for, new dedicated services where potentially frail patients can be referred for further geriatric assessment and management [35,36,37]. In these sites, routine CFS screening is still being actively encouraged and has been expanded to other tumor sites, though uptake by individuals remains variable. At the third site (Cambridge), there was good initial engagement, and some individuals continue to use CFS, but it is no longer being actively prompted, and dedicated frailty services/pathways have not yet been established.

### 3.2. Participants

Ten clinicians were interviewed across the three sites between 23 January and 7 May 2024 (Table 1). Interviews lasted between 40 and 60 min (mean 50 min). A range of clinicians were represented. All participants worked within the lung cancer team, other than two AHPs who worked within the site frailty service, which accepted referrals from the lung cancer team. The time participants had spent working within/alongside the lung cancer team since the CFS piloting project began ranged from 3 months to 5 years (i.e., since inception in 2018). Most had some experience of using CFS, and half of the participants reported using frailty assessments routinely.

Four main themes were generated around the use of frailty assessments in oncology, with associated sub-themes (Table 2). Themes (A–D) and sub-themes are presented with illustrative quotes, identified by the interviewee number (P1–10) and staff role to provide valuable contextual information. Supplementary quotes are provided [Supplementary Table S1].

### 3.3. A. Assessing Fitness and Frailty

**Figure 1:**
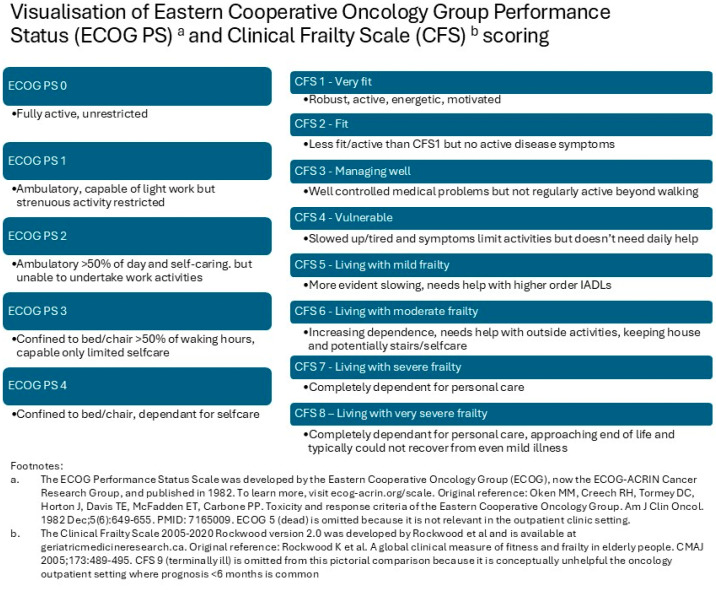
Visualisation of the ECOG Performance Status [36] and Clinical Frailty Scale [13] scoring systems. *Note: the ECOG Performance Status Scale circulates in the public domain and is therefore available for public use. Permission was granted by K Rockwood and team to use/adapt CFS within this figure*.

Clinicians described their experiences and perceptions of how fitness and frailty are assessed in oncology, via both routine assessments, which all patients undergo, and frailty-specific assessments, which can supplement them. A side-by-side comparison of the scores and scoring descriptors for the two key assessment scales referred to (the Eastern Cooperative Oncology Group [ECOG] performance status and the clinical frailty scale [CFS]) is provided in Figure 1.

#### 3.3.1. A.1 Routine Oncology Assessments

Participants described what routine assessment of health and fitness in oncology involves, and the central role of assessing functioning with performance status (PS; Figure 1), which has direct implications for treatment access and is therefore integral to SACT decision-making.

*“the assessment begins when you call them in from the waiting room… then you obviously take their history, and discuss symptoms, discuss comorbidities, medication and then spend a bit of time looking at performance status”*

P01 (oncologist)

Clinicians reported PS to be familiar and simple to use, but felt it to be subjective and lacking granularity, particularly for assessing the complex needs of frail and older patients. Furthermore, they highlighted that cut-points for treatment access (e.g., guidelines/funding criteria citing the requirement to be PS 0–1 to access many treatments, including novel treatments such as immunotherapy) can introduce scoring bias.

*“it doesn’t really capture the complexities of what people are able to do”*

P02 (CNS)

*“[clinicians can] go one way or the other depending on whether they want to give treatment… if you’re a PS2 that comes into clinic, I’m sure there are clinicians that would give them the benefit of the doubt because they actually think the immunotherapy’s better tolerated than the chemotherapy”*

P10 (oncologist)

#### 3.3.2. A.2 Frailty-Specific Assessments

Some participants supplemented routine assessments of fitness with frailty-specific assessments. CFS (Figure 1) was the initial assessment tool most widely used by participants and is the primary focus of the rest of this analysis. CFS was felt to provide a more objective, in-depth assessment than PS and was valuable for guiding decision-making around cancer treatment, but uptake remained variable.

*“I think it [CFS] captures kind of, it fills the gaps in the performance status… I think it helps to guide a consultation and then it helps in the MDT (multi-disciplinary team) for decision-making”*

P02 (CNS)

Clinicians highlighted the value of specialist services providing more in-depth assessments and interventions for patients identified as potentially frail during their cancer care. As highlighted by the mapping exercise, such services had been developed at two sites (The Christie and Newcastle) since the start of the SCFN pilot. Both services offer more comprehensive, multi-domain geriatric assessments and geriatric assessment-driven interventions, personalised to the patients’ needs. The service at the Christie is multi-disciplinary and accepts referrals based on clinical concern, whereas the service in Newcastle is led by a therapy team and has CFS 5+ (which indicates moderate, or more advanced, frailty; see Figure 1) as a mandatory referral criterion. A more detailed description of these services is beyond the scope of this analysis.

### 3.4. B. Clinical Frailty Scale Scoring and Interpretation

Participants described their views and experiences of CFS and reflected on its practicality and value in oncology settings, including how it compares to performance status.

#### 3.4.1. B.1 Ease and Relative Yield

Many found CFS to be easy, simple, and quick to perform, based on information they were already collecting. CFS was felt to provide a better overall assessment and allowed clinicians to summarise and convey important complex information about patient fitness succinctly. However, staff generally had less experience with CFS than with PS. Some found it harder to remember and/or interpret, and others reported that they needed to ask the patient extra questions, and it took longer.

*“[CFS is] fairly self-explanatory, and because all of the numbers come with a… summary for each number, it was fairly easy to do”*

P02 (CNS)

*“I don’t use them routinely, for the reason that I haven’t been able to get familiar with them”*

P04 (oncologist)

#### 3.4.2. B.2 Granularity and Clinical Utility

Most participants reported that CFS was more objective and provided more granularity than PS, helping to better define fitness and guide treatment decisions, particularly for ‘borderline’ (PS 1–2 or 2–3) patients.

*“there’s historically been a bit of a woolly area between [PS] two and a three… [CFS] has allowed us to kind of better define people’s fitness for treatment and who we should be putting forward”*

P02 (CNS)

Participants reflected positively on the prognostic value of CFS, and participants at one site mentioned local published data [15] providing objective evidence to support treatment decisions. CFS scores of 5 or above often prompted particular concern that systemic therapy may not be in a patient’s best interests.

*“[patients with CFS scores] of 5 or above have a very poor prognosis in terms of systemic treatment and that influences our recommendations”*

P07 (respiratory physician)

#### 3.4.3. B.3 Contextual Interpretation

Participants highlighted key considerations for scoring and interpreting CFS. They suggested that it is imperative to consider the patients’ baseline and what is contributing to their CFS scores. It was particularly important to consider the reversibility of contributory factors and whether patient fitness could be improved, such as with pain management or indeed by treating the cancer. Similarly, it was suggested that some long-term physical disabilities can lead patients to score for frailty without having a significant impact on their physiological reserves and tolerance of treatment. Ultimately, scores must be considered in the wider clinical context.

*“you always have to [consider], what was their baseline and… what’s causing their decreased performance status or frailty, you know, is it pain?… can we try and optimise?”*

P08 (CNS)

Participants suggested factors that could impact patient self-reporting, such as cognitive impairment or wanting to be perceived as fit enough to receive treatment. They highlighted that family input can help obtain a fuller and more accurate assessment.

*“they’re saying what they do and their loved one’s behind them going, ‘absolutely not’… so it’s quite handy to, you know, have them there and you get maybe a slightly more realistic picture”*

P08 (CNS)

### 3.5. C. Role of Frailty and Impact of Assessment

Participants discussed their views and experiences around the role of frailty assessments within oncology and highlighted four main ways they can positively impact care.

#### 3.5.1. C.1 Enhancing Communication and Shared Decision-Making with Patients

Participants described how frailty assessments, particularly CFS, can foster a shared understanding about patient fitness and provide a platform for exploring patient priorities and having an open and honest discussion about the intended benefits and potential risks of treatment. Ultimately, this helps to manage expectations and allows patients to make truly informed decisions about their care, taking into consideration what really matters to them. Where assessments indicate treatment may not be in a patient’s best interests, discussing functioning and frailty can increase confidence in a decision to focus on supportive care and quality of life.

*“if you say ‘look, if we take into account this score, it means that the risk of toxicity or side-effects is even higher than what we expected’, so that may help patients to feel confident and comfortable with that decision if they want to avoid chemotherapy and focus on the supported therapy, or the opposite”*

P05 (oncologist)

Participants highlighted the value of positive framing when discussing frailty assessments with patients, and emphasising *‘this is about enabling us to do the best by you’* P02 (CNS)

#### 3.5.2. C.2 Supporting Clinical Treatment Decisions

Participants reported that frailty assessments provide a pause for thought and reflection about the best course of treatment and can support decision-making both in MDT meetings and clinics, particularly for ‘borderline’ (PS 1–2 or 2–3) patients. They suggested scores could help support decisions about commencement of systemic therapy, including regimen, dose, and timing, and have a role in dynamic monitoring of patients, detecting functional decline, and informing changes to the treatment plan.

*“it’s [CFS] definitely beneficial for us to decide on the treatment, not just the treatment itself but also when we should start the treatment and you know, like the dose and everything”*

P09 (oncologist)

#### 3.5.3. C.3 Facilitating Person-Centred Care and Support

Participants reported that, at a basic level, frailty assessment fostered a more person-centred approach.

*“you dig more into the patient’s condition… you are really focusing on them and not only on the investigations… some families appreciate that”*

P05 (oncologist)

Participants highlighted a number of specific ways that frailty assessments improved care beyond their influence on decision-making about anti-cancer treatment. Identification of frailty also helped clinicians recognise vulnerabilities and allowed oncology teams to provide closer monitoring and extra support, begin timely advanced care planning, and seek input from the wider multi-disciplinary team, including palliative care and AHPs (e.g., therapists, dieticians) where appropriate.

*“when patients are more frail I think it’s telling us that we need to be looking at referring to the wider MDT… to AHPs, to palliative care…”*

P03 (AHP)

In some cases, timely detection, and management of potentially reversible frailty-related issues (e.g., polypharmacy, nutritional deficits) could improve patient symptoms and fitness and potentially the tolerance or feasibility of systemic therapy. However, this benefit is less likely to occur where timely access to dedicated frailty services is not possible.

*“[optimisation] has to be done as quick as possible because otherwise you may miss the boat”*

P05 (oncologist)

#### 3.5.4. C.4 Streamlined Care and System-Level Benefits

Clinicians described how improvements in patient care from a frailty-informed approach, including streamlined pathways and better symptom management, care planning, and support, can also have service-level benefits. For instance, appropriately avoiding the need to see an oncologist where high frailty levels indicate systemic therapy is not in a patient’s best interests, or reducing unplanned contact with oncology services (e.g., helpline calls, acute admissions) where there has been proactive symptom management, advance care planning, and community support. Clinicians described significant strain on overstretched clinical staff and systems, which makes such system-level benefits important but difficult to deliver.

*“[if CFS is high] it may be that we give a strong recommendation against systemic treatment and they are managed entirely by respiratory medicine and remotely by the MDT rather than meeting an oncologist”*

P07 (respiratory physician)

*“[after identifying frailty-related issues] we have got them engaged elsewhere. And they have taken up less of the parent oncology team time then because their symptoms changed”*

P06 (AHP)

### 3.6. D. Barriers and Facilitators to Implementation

Participants discussed the practicalities of doing frailty assessments and delivering frailty-informed care, and highlighted key challenges that need to be overcome and enablers to implementation. These are divided into four main areas.

#### 3.6.1. D.1 System-Level Factors

Key system-level barriers reported included a lack of frailty guidance and pathways for onward referral, as well as a lack of awareness of existing pathways/services for the optimisation of frail patients. Clearly documented local consensus on how to assess frailty within current pathways (which tool to use, when, and how), developed with stakeholder input, and improved access to frailty services, were suggested to facilitate uptake.

*“[people ask] ‘where’s the pathway, how do I pick which one [frailty assessment] to do’… there’s no standard practice”*

P06 (AHP)

Integration of frailty assessments into local systems and IT infrastructure (e.g., 2-week wait forms, MDT proforma, and/or EHR) was also suggested to aid completion, improve access to and visibility of scores, and generate data to further support their use.

*“the advantage of having it recorded in a recognised way is that it is accessible to other people and we used it”*

P07 (respiratory physician)

#### 3.6.2. D.2 Exposure, Culture, and MDT Approach

Deeply ingrained individual and team practices and culture around the use of performance status, and a lack of exposure to frailty assessments, were key barriers to incorporating frailty assessments into routine practice. A team-wide approach, local champions, and visibility in clinical areas (e.g., CFS posters, EHR recording) can promote and support day-to-day use.

*“it’s all very well with one person doing a frailty assessment, but it needs to be adopted by the treating team”*

P01 (oncologist)

*“you see people around you using them, that makes it easier… [and] once you use it several times it’s easier and faster”*

P05 (oncologist)

#### 3.6.3. D.3 Time, Resources, and Practical Constraints

Time, staffing, and resources are regularly cited as barriers to the implementation of frailty assessments, particularly in the busy oncology outpatient clinic setting. In this context, participants suggested that to facilitate uptake, frailty assessments should be implemented in a way that minimises the burden on busy staff as much as possible, for example, utilising quick, easy, simple tools, completed by staff other than oncologists, or patient-reported measures.

*“if you are in a rush, if it’s a busy clinic, it’s tempting to not perform those assessments”*

P05 (oncologist)

*“if patients were to fill out something, you know, in the waiting room before they came in and then that could be looked at and any queries clarified by the clinician… that might be useful.”*

P10 (oncologist)

#### 3.6.4. D.4 Evidence and Education

Whilst some clinicians had successfully implemented CFS within their practice without any formal training, others reported a perceived lack of training and knowledge as key barriers to frailty assessment use. It was suggested that education and guidance would help equip staff to assess frailty and use assessments in practice to support decision-making and care. Some clinicians reported a desire to be presented with evidence, including data on tool validity and prognostic value, to help them interpret what scores mean for patients. They also suggested that seeing evidence and example cases demonstrating the benefits of assessing and optimising frailty could promote use.

*“Oh we didn’t get any [training in CFS], no. I think it’s fairly self-explanatory”*

P02 (CNS)

*“I think it may just be a case of education and… ‘what’s in it for me?’… ‘What… is it going to tell me about this patient in front of us?’”*

P03 (AHP)

### 3.7. Mapping to NPT

Practical recommendations to facilitate the implementation of frailty assessments in routine practice are presented within four domains of implementation activity [34] (Table 3). Each recommendation is mapped to the key themes/sub-themes (1–4).

## 4. Discussion

Our findings suggest that a small upfront cost in terms of time and effort required to learn how to use CFS, and implement it in day-to-day oncological practice, can have important and wide-ranging potential benefits for patients, staff, and the wider system. However, uptake remains variable, and there are key barriers that must be overcome for benefits to be realised at scale. Specific recommendations are provided to help overcome barriers and support implementation (Table 3). The findings can inform the development of practical resources to support implementation and include recommendations on what training and guidance for staff should cover.

Our study provides further evidence of the limitations of ECOG performance status [1,17,37] and builds upon the small number of existing qualitative studies exploring the role of frailty assessments in SACT decision-making [17,37]. Warnock’s [17] exploration of patient and staff perspectives on frailty and the role of clinical frailty scale assessments in the treatment for older people with lung cancer focused on key challenges to using frailty assessments to support decision-making, including difficulties defining frailty and discussing it with patients. Our focus on clinician experiences across three diverse sites allowed us to build upon Warnock’s work and study the benefits, barriers, and facilitators of implementation in greater depth. Whilst recognising the challenges, clinicians in our study highlighted the relative yield and value of CFS, which can fill the gaps in performance status and help differentiate between patients considered ‘borderline’ (PS 1–2 or 2–3), and identify patients requiring further optimisation and support. They also suggested that detecting and discussing frailty can support a more person-centred approach and honest, personalised discussions about treatment risk, which is important for truly informed decision-making. On this note, clinicians highlighted the value of positive framing when discussing frailty assessments with patients, which has been highlighted elsewhere [37,38], and emphasised the role of assessments in providing the best possible care and support for each individual, which may go some way to overcoming the challenges identified by Warnock et al. [17]. Sutton et al. [37] also explored both clinician and patient perspectives on the use of a different frailty tool, the electronic Frailty Index (eFI), to support decision-making around SACT. They concluded eFI to be an acceptable addition to SACT decision-making and, similar to our findings, highlighted the importance and value of communicating assessments with patients, and suggested that assessments must be considered within the context of each individual situation [37].

Consistent with the qualitative approach, the sample size of ten participants was small compared to quantitative methods, but it held high information power for addressing the specific aims of the study [25]. The sampling strategy used was designed to ensure a range of perspectives were considered, providing a sufficient exploration of the study topics. Clinicians with strong views on the use of frailty assessments, positive or negative, might have been more likely to volunteer for participation, and such self-selection could have influenced the findings. However, the effect of voluntary participation was minimized using purposive sampling. We ensured a full exploration of the impacts, barriers, and facilitators by purposively sampling participants with both low and high levels of frailty assessment uptake, which helps to increase the transferability and relevance of findings to varied clinicians and clinical contexts.

Our study focused largely on the use of CFS to support decisions around systemic therapy in the lung cancer setting, but many of our findings are transferable to other tumour sites and treatment modalities (e.g., radiotherapy). Our findings around scoring and interpretation of CFS, including its ease, granularity, and relative yield, are somewhat tool specific. However, the principle of the value of frailty assessments for supporting streamlined patient-centred care and decision-making, and the importance of contextual interpretation are transferable to other assessment tools and settings [37]. The concept of higher frailty scores (e.g., CFS 5+) raising concern about potential harms of treatment, and supporting clinical decision-making, is consistent with the quantitative data around their prognostic value [3,14,15,16], but the specific frailty score thresholds that warrant concern/modification to treatment will inevitably vary depending on the assessment and clinical scenario. CFS is a clinician-assessed scale focusing primarily on physical functioning, which contributes to its ease; other tools with different methods of administration (e.g., patient-reporting) and encompassing other domains (e.g., nutrition, cognition, polypharmacy) will inevitably have their own unique benefits and limitations, but a detailed comparison of different tools was beyond the scope of this study.

Whilst this study was initially intended to focus on the use of frailty screening-type assessments within routine oncological care, two of three sites had also established pathways for onward referral of potentially frail patients, allowing a more extended exploration of frailty assessment implementation. To improve the generalisability of the findings, we have attempted to focus this analysis on the role and barriers/facilitators of initial frailty screening assessments in routine oncological care. However, it is inevitably difficult to separate the benefits of initial assessments from those of more comprehensive assessments and further management. Frailty assessments were sometimes discussed in general terms by participants, and whilst the interviewer attempted to clarify the role and impact of initial assessments during interviews, and quotes that clearly focused on more comprehensive assessments were not included in this synthesis, it is inevitable that access to more in-depth assessments contributes to the impacts reported.

In the context of geriatric assessment being increasingly recommended in cancer settings [4,5,6,7], but not widely implemented, our findings support the notion that the implementation of simple frailty assessments may convey some benefits in their own right. However, screening assessments should be considered in the wider clinical context and ideally be supplemented by more comprehensive, multi-domain assessments with optimisation of deficits as recommended in international guidance [4,5,6,7]. As demonstrated at two of the three sites [39,40], the implementation of simple frailty assessments can provide an important step towards providing more personalised frailty-informed care and the development of specialist frailty/onco-geriatric services to provide more comprehensive assessment. More research is needed to explore the benefits of simple frailty assessments in the absence of access to specialist frailty/onco-geriatric services, and within different models of care. Which patient outcomes are most important when measuring benefit is much debated [41]; findings from this study could support the selection of outcomes for evaluating frailty-informed care (e.g., measures of decision quality/satisfaction, interventions to optimise frailty, including referral to wider MDT, advanced care planning, unplanned care utilisation, goal-aligned care). As well as demonstrating benefits for individual patients, our findings also highlight important potential system-level benefits, such as streamlining of patient pathways and reduced acute/unplanned care use, which should be further explored.

It is important to consider the role of the researchers and how their positionality may have influenced the data collection and interpretation. The lead researcher [JP] is an oncologist with an interest in frailty and inevitably has pre-existing views and experiences. The input of the wider research team, from a range of clinical and academic backgrounds, helped to provide balance to the data collection and analysis, and the use of a second coder further aided reflexivity and helped ensure the findings presented reflect the staff's experiences and views. Patient interviews were not undertaken in this study, but the wider FRAME project Patient Advisory Group involvement ensured patient perspectives were considered during study design and analysis, and patient interviews are planned in future work [18].

Our specific recommendations are intended to facilitate frailty assessment implementation in practice, helping to bridge the knowledge gap between international best practice and the reality of routine care implementation. We highlight the need to develop and promote pathways, guidance, and training around the assessment and management of frailty locally. We suggest integrating frailty assessments into IT systems to increase accessibility and allow data generation and analysis, which can be used to demonstrate local need and help build a business case to fund frailty service development. We also emphasise the importance of an MDT approach and highlight the important role local champions have in promoting frailty assessment uptake by developing pathways and services whilst collecting and sharing evidence of benefit to drive improvements in care. The findings of this study can inform the development of new practical interventions to support the use of frailty assessments in practice, and have already guided the creation of patient materials and a series of scripts for clinicians to support frailty-informed shared decision-making in the clinic as part of the FRAME (18) intervention development project.

## 5. Conclusions

Five years after the set-up of a national pilot implementing frailty assessment via the CFS in oncology clinics, uptake remains variable. Clinicians highlight the simplicity of CFS and its additional value for assessing patients considered borderline according to performance status. However, significant barriers to implementation persist, including awareness of and access to guidance and training, and practical constraints. With growing evidence of the benefits of frailty-informed approaches, these should become standard care, and we provide practical recommendations and strategies to support the routine implementation of frailty assessments, which is an essential step towards delivering optimal personalised cancer care for our aging population.

## Figures and Tables

**Table 1:** Interviewee demographic data.

Demographic Factor	Results	No of Participants
Site	Cambridge	3
	The Christie	4
	Newcastle	3
Age group	30–39	5
	40–49	3
	50–59	1
	60–69	1
Ethnicity	White British	8
	White Other	1
	Asian or Asian British	1
Profession	Medical Oncologist	3
	Clinical Oncologist	2
	Clinical Nurse Specialist	2
	Allied Health Professional	2
	Respiratory physician	1
Grade	Registrar	3
	Consultant	3
	NHS Agenda for Change ^1^ Band 6	1
	NHS Agenda for Change ^1^ Band 7	2
	NHS Agenda for Change ^1^ Band 8	1
Self-reported frailty assessment uptake ^2^	High/moderate (response: always, often, or sometimes)	5
	Low (response: never or rarely)	5
Frailty assessments used ^3^	Clinical Frailty Scale	7
	Geriatric-8	2
	Comprehensive Geriatric Assessment	1
	N/A	2

^1^. NHS Agenda for Change banding is used to categorise the grades of most healthcare staff (excluding doctors) in the UK. For clinical roles, broadly, band 6 indicates an experienced/specialist clinician, band 7 indicates an advanced specialist clinician, and band 8 indicates senior leaders and consultant-level specialist roles. ^2^. Question wording: *“How often do you use a frailty assessment when assessing a **new patient (any age)** who is for consideration of systemic anti-cancer treatment?”*. ^3^. Question wording: *“Which frailty assessments/tools have you personally used (if any)?”.*

**Table 2:** Summary of key themes and sub-themes.

Theme	Sub-Theme
**A. Assessing fitness and frailty** Captures how routine and frailty-specific assessments are conducted and perceived in oncology.	A.1 Routine oncology assessments
	A.2 Frailty-specific assessments
**B. Scoring and interpreting the CFS** Explores how CFS is understood and used in practice.	B.1 Ease and relative yield
	B.2 Granularity and clinical utility
	B.3 Contextual interpretation
**C. Role of frailty and impact of assessment** Highlights key ways in which frailty assessments can influence care.	C.1 Enhancing communication and shared decision-making with patients
	C.2 Supporting clinical treatment decisions
	C.3 Facilitating person-centred care and support
	C.4 Streamlined care and system-level benefits
**D. Barriers and facilitators to implementation** Identifies factors that help or hinder the implementation of frailty assessment and subsequent frailty-informed care.	D.1 System-level factors
	D.2 Exposure, culture, and MDT approach
	D.3 Time, resources, and practical constraints
	D.4 Evidence and education

CFS = clinical frailty scale. MDT = multi-disciplinary team.

**Table 3:** Practical recommendations to facilitate frailty assessment implementation in routine practice.

Implementation Activity Domain [34]	Practical Recommendations for Implementation (Mapping to Themes/Sub-Themes A–D)
Information Strategies (what do staff need to know to contribute to implementation?)	Staff should be provided with/signposted to training and guidance to provide them with the knowledge and skills to implement frailty assessments. Training/guidance should cover:
	Which frailty assessment to use (a recommendation or information to help them decide; D.1, D.4). How to undertake the frailty assessment of choice (who completes it, when in the pathway, and how; D.1, D.4). What scores mean for patients and how they can/should be used in practice (B.1–B.3, C.1–C.3, D.1, D.4). The validity and benefits of the assessment (for patients, staff, and the system as a whole), signposting to evidence which may come from local cases, data, or publications (B.1–B.2, C.1–C.4, D.4). Local pathways for onward referral if/when frailty is identified, e.g., referral criteria, processes (D.1, D.4).
Empowerment Strategies (what needs to be accomplished to equip staff to participate in implementation?)	Staff should be empowered and equipped to participate in implementation by:
	Their involvement, as key stakeholders, in the development of local frailty guidance, pathways, and services to ensure the agreed processes (who does what, when, and how) work for staff and in the local context (D.1). Promotion of training and guidance on frailty assessment and optimisation (D.1, D.4; see Information strategies above).
Service User Strategies (how can service users contribute to implementation?)	Healthcare teams can involve patients and their loved ones in the implementation of frailty assessments by:
	Inviting them to contribute to the assessment, for example, by using frailty assessments that utilise patient self-report and getting family input (B.3, D.3). Framing frailty assessment in a positive way, highlighting the focus on helping ensure patients receive the right treatment and support, and ultimately the best possible care (C.1). Using information from frailty assessments within consultations to facilitate open and honest personalised discussions with patients about the risks of treatment, elicit patient priorities, and help manage expectations during the shared decision-making process (C.1). Using frailty assessments to identify patients who are likely to benefit from additional support or could be optimised to improve their treatment options or tolerance of treatment (C.3)
	Patients can also contribute to implementation through their involvement as key stakeholders, alongside staff, in the development of local consensus/guidelines/pathways for the assessment and management of frailty (D.1).
Leadership Strategies (what do leaders need to do to promote implementation?)	Local champions and leaders have a key role in promoting and supporting the successful implementation of frailty assessments in routine care. Local champions, supported by management, should seek to promote implementation by:
	Establishing local consensus/guidelines/pathways for the assessment and management of frailty, taking account of stakeholder views, to ensure a cohesive, pragmatic MDT-wide approach which minimises burden on busy teams and clinics as much as possible (D.1–D.3). Integrating frailty assessments into pathways and electronic health records to promote score documentation, visibility, and accessibility (D.1). Collecting and promoting evidence of the benefits of frailty assessment and management using data and case examples, both to encourage routine implementation and support business cases to secure funding and staff to develop/deliver frailty pathways and services (C.1–C.4, D.1, D.3, D.4). Developing and/or promoting training and guidance around the assessment and management of frailty (D.1, D.4). Developing, promoting, and/or increasing access to pathways for onward referral and optimisation of patients identified as frail (D.1).

## Data Availability

The datasets presented in this article are not readily available to maintain participant anonymity. Requests to access the datasets should be directed to j.pearce@leeds.ac.uk.

## Supplementary Materials

The following supporting information can be downloaded at: https://www.mdpi.com/article/10.3390/cancers18050884/s1, Supplementary Table S1: Full list of exemplary quotes.

## Abbreviations

The following abbreviations are used in this manuscript:

AHP	Allied Health Professional
CFS	Clinical Frailty Scale
CNS	Clinical Nurse Specialist
ECOG	Eastern Cooperative Oncology Group
EHR	Electronic Health Record
FRAME	Frailty-infoRmed cAncer ManagemEnt
MDT	Multi-disciplinary Team
MIND-IT	Making Informed Decisions Individually and Together
NPT	Normalisation Process Theory
PS	Performance Status
SACT	Systemic Anti-Cancer Treatment

## Appendix A

### Appendix A.1. Further Details on Sampling Strategy

A purposive sample of 10–12 HCPs was sought in line with the following inclusion criteria.

- Healthcare professional with at least 3 months of experience working within a project/service where frailty assessments have been implemented
- A professional role involves undertaking frailty assessments and/or subsequent SACT treatment decision-making (though may not routinely undertake frailty assessments within their own practice)

There were no exclusion criteria.

To maximise the value of the data for addressing the study aim and objectives, the following two purposive sampling techniques were combined [25]:

- Stakeholder sampling based on professional role (oncologist, specialist nurse) to ensure that participants represent the main healthcare professionals involved in assessing frailty and utilising the information from assessments within cancer treatment decision-making
- Maximum variation purposive sampling based on the following criteria to ensure that a diverse range of perspectives is considered:
  ○ The organisation and team they are working in (hospital site and tumour site)
  ○ Years of experience working in the current professional role
  ○ Adoption of frailty assessments in routine practice (low vs. high adopters)

## Appendix B

### Appendix B.1. Sample Topic Guide

This is a sample topic guide for interviewing healthcare professionals. This guide will be updated and reviewed during the interview process to target emerging themes and new topics of interest.

Welcome and study information

Thank you for agreeing to help with this research. The overall study aim is to develop frailty-informed cancer management (FRAME) intervention to support shared cancer treatment decision-making between patients and their medical teams. In this interview study we are trying to find out about healthcare professionals experiences of assessing frailty and making systemic anti-cancer treatment decisions with patients in oncology, to help inform the development of the FRAME intervention.

Everything that you say during this interview is confidential, with the usual caveat of confidentiality only being broken if there something is disclosed which raises a serious concern of risk of harm. It is possible that some of the things you say during the interview will be quoted in research publications. If that happens, the quotes will be anonymous—which means that we will not use your name or say anything about you that would make you identifiable.

I am going to ask you to talk about your experiences and views on the use of frailty assessments and decision-making in cancer care, with a specific focus on what happens around the time of the first oncology clinic appointments for patients being considered for systemic anti-cancer treatment.

There are no right or wrong answers to any of the questions. As we go along, if there are any questions that you would rather not answer, this is fine, just let me know and we will move on. We can stop the interview at any time, and if you change your mind about taking part, just let me know at any point.

Is there anything you want to ask me about?

Can I just make sure that the details I have about you are correct?

Check name and demographic details

I am going to turn on the Dictaphone now if this is OK with you?

Introduction

Please can you talk me through how a new patient is assessed when an initial systemic anti-cancer treatment plan is being made in your centre? What is your role in this process?

Research Topics to cover:

PART A: Experience and views of health and fitness/frailty assessment within cancer care

PART B: Experience and views of systemic anti-cancer treatment decision-making

PART A: Experience and views of health and fitness/frailty assessment within cancer care

**Describe in a little more detail how you assess patients during treatment planning**

**Do you think frailty assessments have a role when assessing patients in oncology?**

**Do you personally use frailty assessments?**

If no:

Why not?

What other information/tools do you use? How documented? Strengths/limitations?

If yes:

Which? When/where are they undertaken? By whom? How documented?

Are they helpful?/What impacts (positive or negative) do they have on patient care (if any)?

E.g., impacts on (1) the consultation (2) cancer treatment decision/planning (3) frailty-related issue management (4) involvement of wider MDT/onward referral (5) advanced care planning

If no impact, why?

**Do you think frailty assessments (have potential to) add value on top of standard oncological assessments (history/exam, PS assessment etc)?**

**What things do/could support or prevent you from using frailty assessments?**

**Do you talk to patients about their fitness/frailty (and likely impact on treatment)?**

If yes, how? How valuable is this?

If no, why not? Would you consider doing so?

PART B: Experience and views of systemic anti-cancer treatment decision-making

**How do you/your team go about making/supporting a decision about SACT?**

**How (if at all) do you involve patients and their family/carers in decision-making?**

What influences the way in which you involve them? E.g. age, life situation

Do you provide any materials (e.g., leaflets) to support their understanding/decision?

Can you give any examples of times you (or colleagues) have involved patients/families in decision-making well/less well?

**What things do/could support or prevent you from involving patients and their families in decision-making?**

There is a framework called MIND-IT (making informed decisions individually and together) which provides a diagrammatic representation of how shared decisions are made between patients and healthcare professionals, perhaps we could look at this together to brainstorm thoughts? [Show MIND-IT]. Does this prompt ideas of things that do/could support shared decision-making? e.g., information or training materials, guidance, systems/cultural changes… Anything else?

**Do you think that frailty assessments could support shared decision-making?**

If yes—why/how?

If no—why not?

If this is something you already do, can you give any examples?

Closing question

Do you have any other thoughts on how frailty assessment and/or shared decision-making within oncology can be improved?

Summary, thanks, debrief and close
