# Supplementary material for: Clinicians’ Experiences of Implementing Clinical Frailty Scale Assessments in Lung Oncology Clinics: A Qualitative Interview Study
Source: Cancers (Basel). 2026 Mar 9;18(5):884. doi: 10.3390/cancers18050884 (PMC12984377; doi:10.3390/cancers18050884)
Supplement: Supplementary file 1 [file cancers-18-00884-s001.zip › cancers-4137572-supplementary.pdf]

Supplementary table S1. Full list of exemplary quotes

Theme / Sub-theme	Exemplary quotes
**A. Assessing fitness and frailty:** Captures how routine and frailty-specific assessments are conducted and perceived in oncology.	
A.1 Routine oncology assessments	"you call them in from the waiting room and you watch how they get up from the chair and how, you know, how well they're walking into the clinic and if they need a wheelchair, walking stick... then you obviously take their history, and discuss symptoms, discuss comorbidities, medication, and then spend a bit of time looking at performance status, so what is, you know, what are their activity levels, what's their day-to-day life consist of. Exercise tolerance, and then it's indicated on physical examination, but not always, and then looking at their blood tests" P01 (oncologist)
	"[with PS] I obviously have an internal feedback loop, I see how that patient does and that informs me, on that patient and future patients … who might benefit from treatment and who might not, and I don't have that sort of reference point with the other scores" P04 (oncologist)
	"it [PS] doesn't really capture the complexities of what people are able to do" P02 (CNS)
	"PS] can be very subjective, can't it, sometimes and what you think and what someone else thinks are completely different" P08 (CNS)
	"it's almost not spoken about… most treatments in terms of SACTs with NICE or a cancer drugs fund labels on them will say, 'is the patient performance status 0 or 1' and… I certainly wouldn't want wildly to change it [their PS scoring], but if it's sort of, you know, one way or the other, you would certainly err on the benefit of the doubt to access a treatment that they thought that they might benefit from" P04 (oncologist)
	"[clinicians can] go one way or the other depending on whether they want to give treatment… if you're a PS2 that comes into clinic, I'm sure there are clinicians that would give them the benefit of the doubt because they actually think the immunotherapy's better tolerated than the chemotherapy" P10 (oncologist)
A.2 Frailty-specific assessments	"people are doing the CFS but it's not consistent" P06 (AHP)
	"I think it [CFS] captures kind of, it fills the gaps in the performance status… I think it helps to guide a consultation and then it helps in the MDT for decision-making" P02 (CNS)
	"in the past I would be guilty of just making my own assumption based on a limited set of questions whereas now [since implementing CFS] I am better at asking a bit more detail and scoring according to that" P07 (respiratory physician)
	"[the frailty team] would complete a really comprehensive initial assessment [which] includes their frailty score obviously… physical function… mental health… socially what's going on… cognition… symptoms… everything" P03 (AHP)
**B. Clinical Frailty Scale scoring and interpretation:** Explores how CFS is understood and used in practice.	
B.1 Ease and relative yield	"[CFS is] fairly self-explanatory, and because all of the numbers come with a… summary for each number, it was fairly easy to do… it doesn't take long… it's part of the information that we're collecting anyway" P02 (CNS)
	"a small number of extra questions taking very little extra time but I think producing a better overall assessment." P07 (respiratory physician)
	"I don't use them routinely, for the reason that I haven't been able to get familiar with them" P04 (oncologist)
	"you need to ask them quite a few questions so it takes longer" P05 (oncologist)
B.2 Granularity and clinical utility	"there's historically been a bit of a woolly area between [PS] two and a three… [CFS] has allowed us to kind of better define people's fitness for treatment and who we should be putting forward" P02 (CNS)

	“it has more granularity I guess and that’s the main thing, that it helps to decide if you are a bit unsure or if you want to produce notes it may, yeah, justify your decision.” P05 (oncologist)
	“[patients with CFS scores of] of 5 or above have a very poor prognosis in terms of systemic treatment and that influences our recommendations” P07 (respiratory physician)
	“data that has been published from just shy of seven hundred patients that shows that [CFS] predicts prognosis… and if we look specifically at lung, if a patient is admitted and they have a frailty score of 6 their medium survival tends to be around about 26 days, which is no amount of time” P03 (AHP)
B.3 Contextual Interpretation	“you always have to [consider], what was their baseline and, I suppose, what’s causing their decreased performance status or frailty, you know, is it pain?… can we try and optimise?” P08 (CNS)
	“there are things, maybe permanent conditions that affect people's ability to do things which don't necessarily give a reflection of their reserves in terms of complications, like long-term amputations or spinal problems” P07 (respiratory physician)
	“they’re saying what they do and their loved one’s behind them going, ‘absolutely not’, you’re like ‘when did you last do that’, so it’s quite handy to… have them [loved ones] there and you get maybe a slightly more realistic picture... because people just want treatment, don’t they, so they’ll just say anything or say what they think they want us to hear” P08 (CNS)
**C. Role of frailty and impact of assessment:** Highlights how frailty assessments can influence care	
C.1 Enhancing communication and shared decision-making with patients	“if you say ‘look, if we take into account this score, it means that the risk of toxicity or side-effects is even higher than what we expected’, so that may help patients to feel confident and comfortable with that decision if they want to avoid chemotherapy and focus on the supported therapy, or the opposite” P05 (oncologist)
	“some of our patients come in, going, ‘Oh yeah the other doctor told me that I’m good, but I’m a bit frail,’ so they prepare the patient to understand that, it’s managing the expectations… and maybe helps them make an informed decision regarding their treatment.” P06 (AHP)
	“I know that the clinical frailty scores aren’t designed for this, but I think it enables us to pull out as well actually what patients’ priorities are as well and kind of better understand what’s important to them. You know, if being able to get washed and dressed in the morning and get out with their grandchildren once a week is what their priority is but they struggle with much beyond that, then maybe anti-cancer treatment isn’t the right thing for them” P02 (CNS)
	“I don’t think we ever tell patients that we’re giving them a number or you know, trying to kind of put them in a box, so to speak. That sounds terrible, but I think it’s more just about you know, I’m going to ask you some questions… and that will kind of guide us with what treatment options you might have, but also what kind of support is available to you… with a positive slant of, ‘this is about enabling us to do the best by you’” P02 (CNS)
C.2 Supporting clinical treatment decisions	“a patient might have been marked down as… performance status 1/2, or 2, and… a clinical frailty score of 6, and that kind of helps to generate further conversation in the MDT meeting about you know, what is it about this patient that gives them a clinical frailty score of six, and should we be thinking about treatments for this patient, or are we likely to do them more harm than good?” P02 (CNS)
	“it’s [CFS] definitely beneficial for us to decide on the treatment, not just the treatment itself but also when we should start the treatment and you know, like the dose and everything” P09 (oncologist)

	"it's [CFS] sensitive to functional change… it informs treatment decisions including when to stop treatment or when to give treatment breaks or when to dose reduce" P03 (AHP)
C.3 Facilitating person-centred care and support	"you dig more into the patient's condition, I felt they actually prefer that because they see that you are really focusing on them and not only on the investigation so the tumour cells or whatever, some families appreciate that" P05 (oncologist)
	"when patients are more frail I think it's telling us that we need to be looking at referring to the wider MDT… to AHPs, to palliative care…" P03 (AHP)
	"it enables you to think about and plan for the future because, you know, people that are at the frailer end of the spectrum are likely to need more in the way of support" P02 (CNS)
	"[the frailty team] may improve patients' fitness and in that case it may be easier or more feasible to go ahead with chemotherapy," P05 (oncologist)
	"[optimisation] has to be done as quick as possible because otherwise you may miss the boat" P05 (oncologist)
C.4 Streamlined care and system-level benefits	"[if CFS is high] it may be that we give a strong recommendation against systemic treatment and they are managed entirely by respiratory medicine and remotely by the MDT rather than meeting an oncologist" P07 (respiratory physician)
	"[after identifying frailty-related issues] we have got them engaged elsewhere. And they have taken up less of the parent oncology team time then because their symptoms changed" P06 (AHP)
	"I think that we're getting less hotline calls with the frail patients who have been assessed by our [frailty team]." P09 (oncologist)
	"[our frailty team has] data about things like reduced length of stay, admission avoidance, huge numbers of future planning conversations" P03 (AHP)
**D. Barriers and facilitators to implementation:** Identifies factors that help or hinder the implementation of frailty assessment and subsequent frailty-informed care.	
D.1 System-level factors	"[people ask] 'where's the pathway, how do I pick which one [frailty assessment] to do'… there's no standard practice" P06 (AHP)
	"[to facilitate frailty assessment and optimisation] have internal protocols and make people aware of that, because even if you write a nice protocol if nobody is using it, it's useless… and try to get lots of feedback before writing those protocols… so again it is streamlined and agreed by everyone" P05 (oncologist)
	"the other thing with frailty is… if they can be medically optimised prior to starting treatment, that might be appropriate… [but] we don't have any oncogeriatrics" P01 (oncologist)
	"the advantage of having it recorded in a recognised way is that it is accessible to other people and we used it" P07 (respiratory physician)
D.2 Exposure, culture and MDT approach	"[barriers to frailty assessments are…] the exposure, mainly… if I was working in a department or in a team that was regularly using them and kind of wanted to know that information, I certainly would use them… it's just currently not really used because we've just been using performance status." P10 (oncologist)
	"it's all very well with one person doing a frailty assessment, but it needs to be adopted by the treating team" P01 (oncologist)
	"you see people around you using them, that makes it easier. … [and] once you use it several times it's easier and faster" P05 (oncologist)
	"[one of the reasons for buy-in at our site is…] we were involved in the first pilot and we were lucky, there were probably two key individuals who were very keen and enthusiastic and… have been very good at keeping it on their radar and that's really why…" P07 (respiratory physician)
	"it would be helpful to have a print out of it [CFS] in the wall… when you're implementing anything new it takes a while to get your head around doesn't it?" P02 (CNS)

D.3 Time, resources and practical constraints	"[for adoption of a frailty assessment into my practice…] I think it would need to be quick and easy" P04 (oncologist)
	"if you are in a rush, if it's a busy clinic, it's tempting to not perform those assessments" P05 (oncologist)
	"if patients were to fill out something, you know, in the waiting room before they came in and then that could be looked at and any queries clarified by the clinician… that might be useful." P10 (oncologist)
	"we've got quite a big specialist nursing team… it doesn't necessarily have to be a clinician doing a frailty assessment" P01 (oncologist)
D.4 Evidence and education	"I think it may just be a case of education and… 'what's in it for me?'… 'what… is it going to tell me about this patient in front of us?'" P03 (AHP)
	"I'd need to be actively educated on how to use it and… if someone said, here's a piece of research that uses the score, as opposed to another score, and these are the outcomes which are significantly better, whatever they may be, then, yeah, that would help persuade me" P04 (oncologist)
	"Oh we didn't get any [training in CFS], no. I think it's fairly self-explanatory" P02 (CNS)
